# Supplementary material for: Comprehensive benchmarking of somatic single-nucleotide variant and indel detection at ultra-low allele fractions using short- and long-read data
Source: bioRxiv. 2025 Oct 14:2025.10.13.681545. Preprint. [Version 1] doi: 10.1101/2025.10.13.681545 (PMC12632906; doi:10.1101/2025.10.13.681545)
Supplement: 1 [file NIHPP2025.10.13.681545v1-supplement-1.pdf]

bioRxiv preprint doi: https://doi.org/10.1101/2025.10.13.681545; this version posted October 14, 2025. The copyright holder for this preprint (which was not certified by peer review) is the author/funder, who has granted bioRxiv a license to display the preprint in perpetuity. It is made available under aCC-BY 4.0 International license.

Supplementary Notes

Quality controls for sequencing data

For each BAM file, standard sequencing quality metrics, such as mapping rates, estimated insert sizes (**Figure S1**), average whole genome coverage, and other alignment-based statistics, were analyzed using Samtools[1] (*v1.17*), Picard[2] (*v3.0.0*), mosdepth[3] (*v0.3.9*), and a custom in-house Python script. For PacBio and ONT data, reads were evaluated to assess the read-length distribution, N50, and other QC metrics specific to long-read data. For Illumina data generated on the NovaSeq platforms, raw reads were evaluated for base quality and base composition, and reads containing poly-G artifacts were removed using FastQC[4] (*v.12.0*). The full description of the data quality control (QC) and assessment at the SMaHT Data Analysis Center is in the SMaHT Benchmark Flagship paper [5].

bioRxiv preprint doi: https://doi.org/10.1101/2025.10.13.681545; this version posted October 14, 2025. The copyright holder for this preprint (which was not certified by peer review) is the author/funder, who has granted bioRxiv a license to display the preprint in perpetuity. It is made available under aCC-BY 4.0 International license.

Supplementary figures

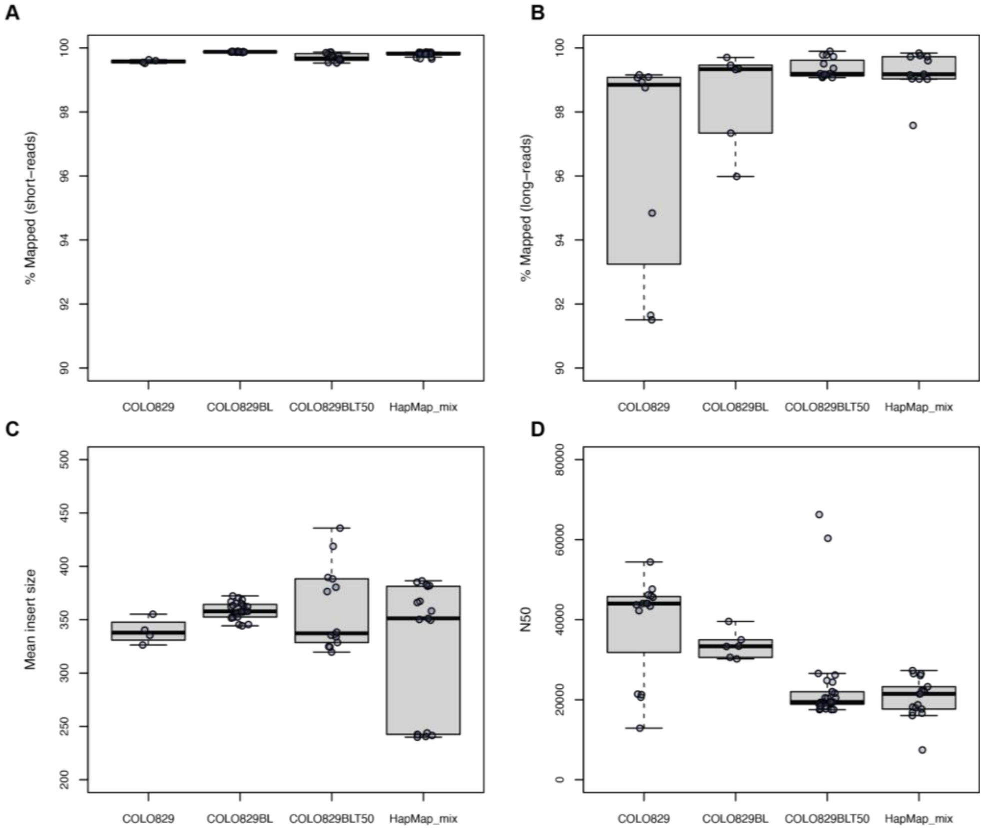

Figure S1. Overall mapping rates of Illumina short-read (A) and PacBio, standard ONT long-read (B) bulk WGS data, as well as estimated insert sizes (C; for short-read WGS) and N50 (D; for long-read WGS) across the benchmark cell line samples analyzed in this study.

bioRxiv preprint doi: https://doi.org/10.1101/2025.10.13.681545; this version posted October 14, 2025. The copyright holder for this preprint (which was not certified by peer review) is the author/funder, who has granted bioRxiv a license to display the preprint in perpetuity. It is made available under aCC-BY 4.0 International license.

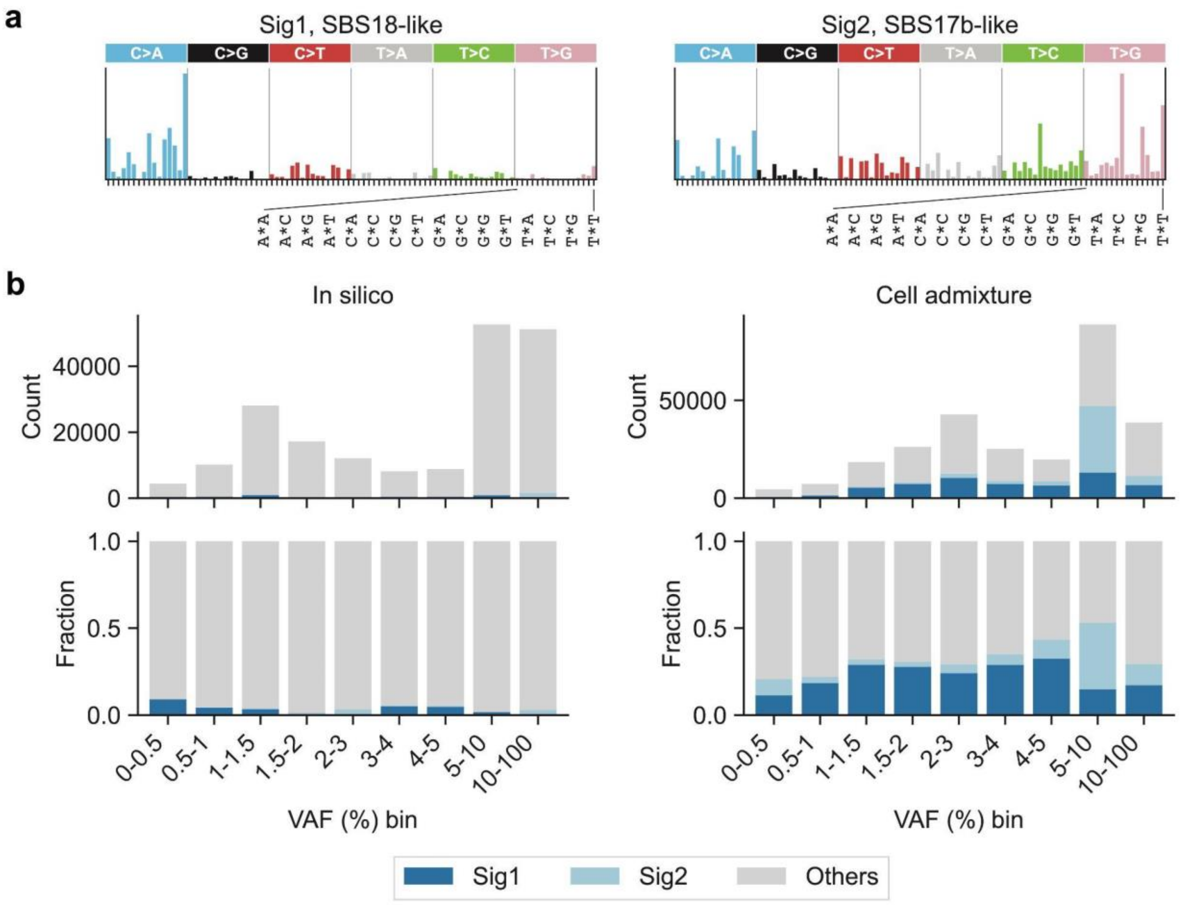

Figure S2. ROS-associated mutational signatures in cell admixture and in silico COLO829BLT50 datasets. (a) Two *de novo* mutational signatures likely associated with ROS damages in cell cultures. Signatures were *de novo* extracted from “nonvariant” false positive SNV calls stratified by sample, VAF bin, and variant calling pipeline. Sig1 (left) is similar to COSMIC SBS18 (cosine similarity = 0.93). Sig2 (right) shows strong peaks at N[T>G]T, especially C[T>G]T, which are characteristics of COSMIC SBS17b. Both SBS18 and SBS17b are annotated as ROS-associated in COSMIC. **(b)** Absolute (top) and relative (bottom) exposures of ROS-associated *de novo* mutational signatures in *in silico* (left) and cell admixture (right) COLO829BLT50 data. Exposures were calculated for “nonvariant” false positive SNV calls pooled over samples and variant calling pipelines.

bioRxiv preprint doi: https://doi.org/10.1101/2025.10.13.681545; this version posted October 14, 2025. The copyright holder for this preprint (which was not certified by peer review) is the author/funder, who has granted bioRxiv a license to display the preprint in perpetuity. It is made available under aCC-BY 4.0 International license.

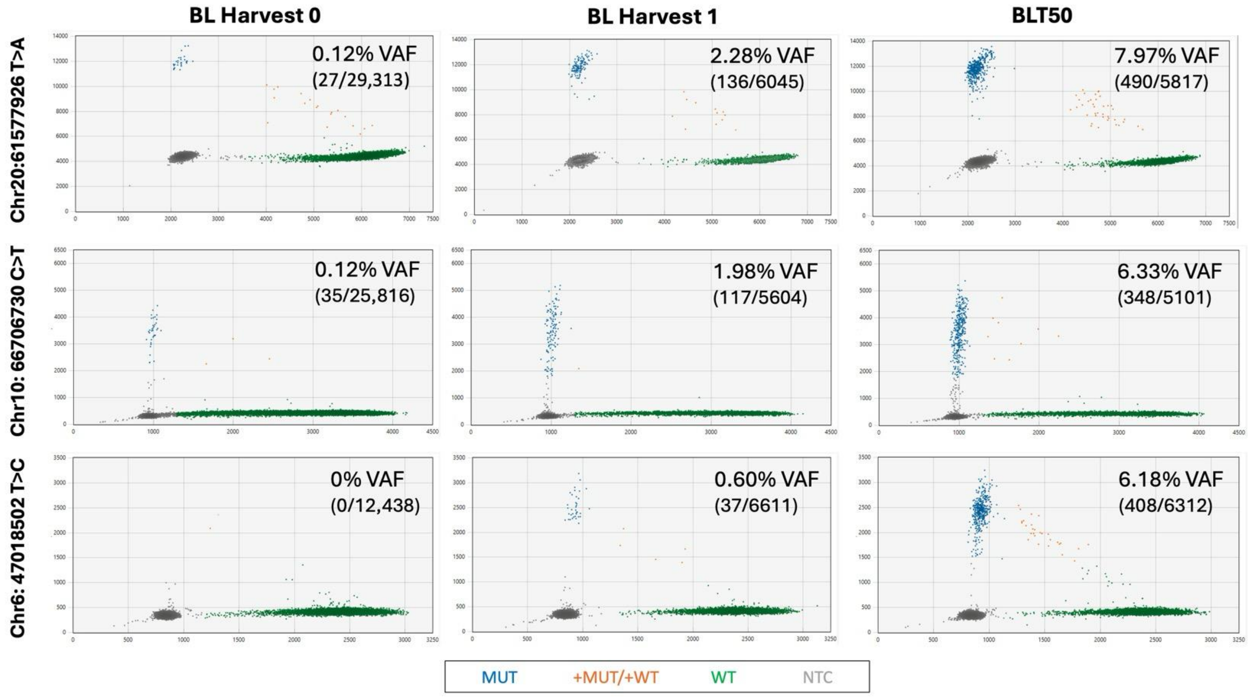

Figure S3. Digital Droplet PCR (ddPCR) adjudication of variants found in the cell-culture derived set. Three variants were selected for ddPCR sequencing. Variant allele frequencies for each sample are displayed in the upper right corner of each panel, along with the droplet counts below. Blue colored droplets indicate the homozygous presence of the variant, and orange colored droplets indicate a heterozygous presence, while green colored droplets show only WT allele at the position. Grey indicates drop out.

bioRxiv preprint doi: https://doi.org/10.1101/2025.10.13.681545; this version posted October 14, 2025. The copyright holder for this preprint (which was not certified by peer review) is the author/funder, who has granted bioRxiv a license to display the preprint in perpetuity. It is made available under aCC-BY 4.0 International license.

Figure S4

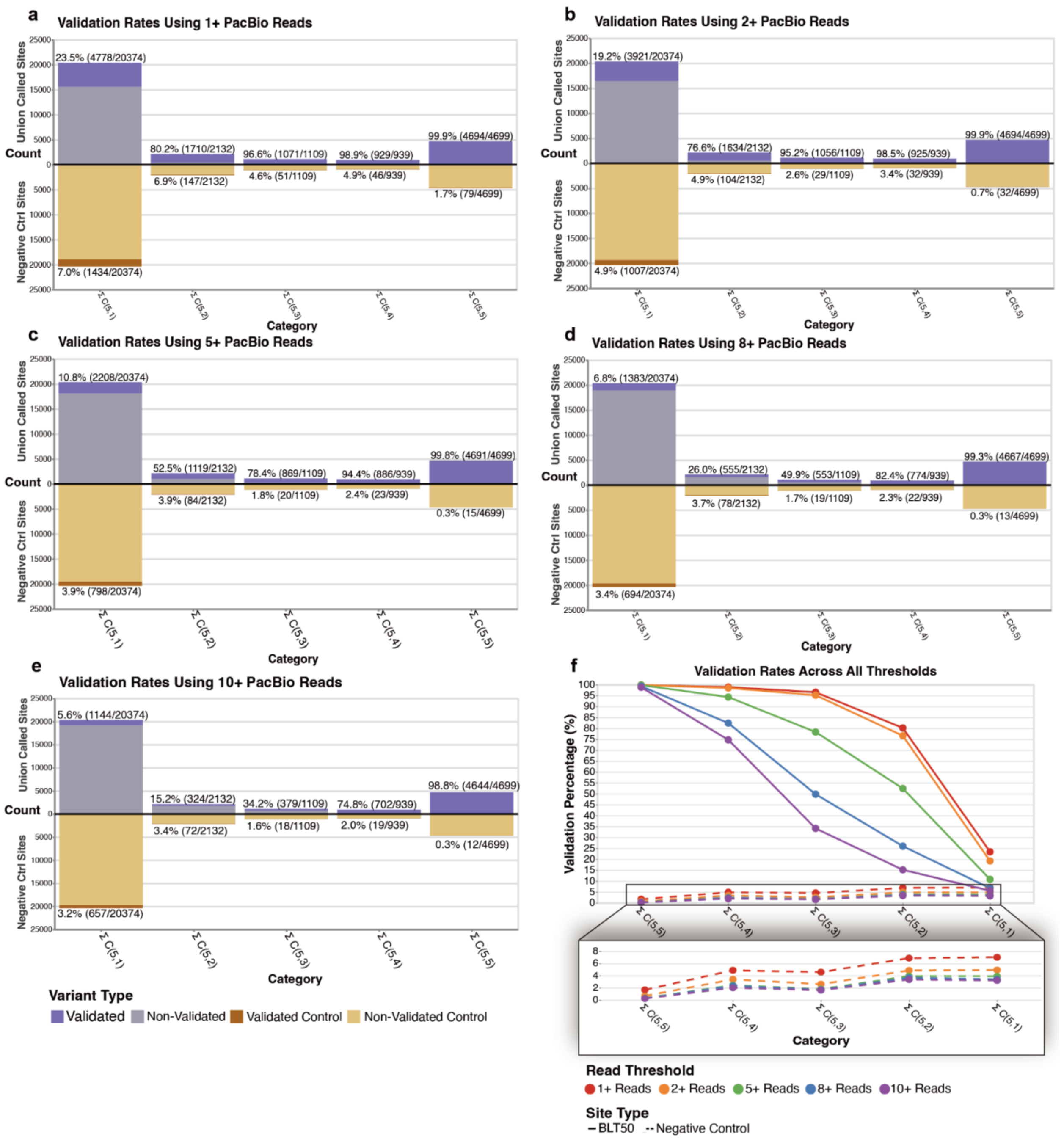

Figure S4. Validation rates for COLO829BLT50 admixture-only variants with increasing long-read coverage thresholds. For each histogram in panels (a-e), the top purple bars denote the counts of candidate and validated admixture-only variants. The bottom tan bars represent the same counts of candidate and validated alleles found in a negative control homogenate tissue which was sequenced using identical preparation methods and technology. Each histogram column represents the variants found in (**a**) single replicate (∑ C(5,1)), (**b**) two replicates (∑ C(5,2)), (**c**) three replicates (∑ C(5,3)), (**d**) four

bioRxiv preprint doi: https://doi.org/10.1101/2025.10.13.681545; this version posted October 14, 2025. The copyright holder for this preprint (which was not certified by peer review) is the author/funder, who has granted bioRxiv a license to display the preprint in perpetuity. It is made available under aCC-BY 4.0 International license.

replicates (∑ C(5,4)), and (**d**) all five replicates (∑ C(5,5)). (**f**) comparison of the validation rates across increasing long-read thresholds, demonstrating that at our selected threshold of 2+, we maximize the amount of admixture-only variants validated, while minimizing the false positives that are also validated in the negative control.

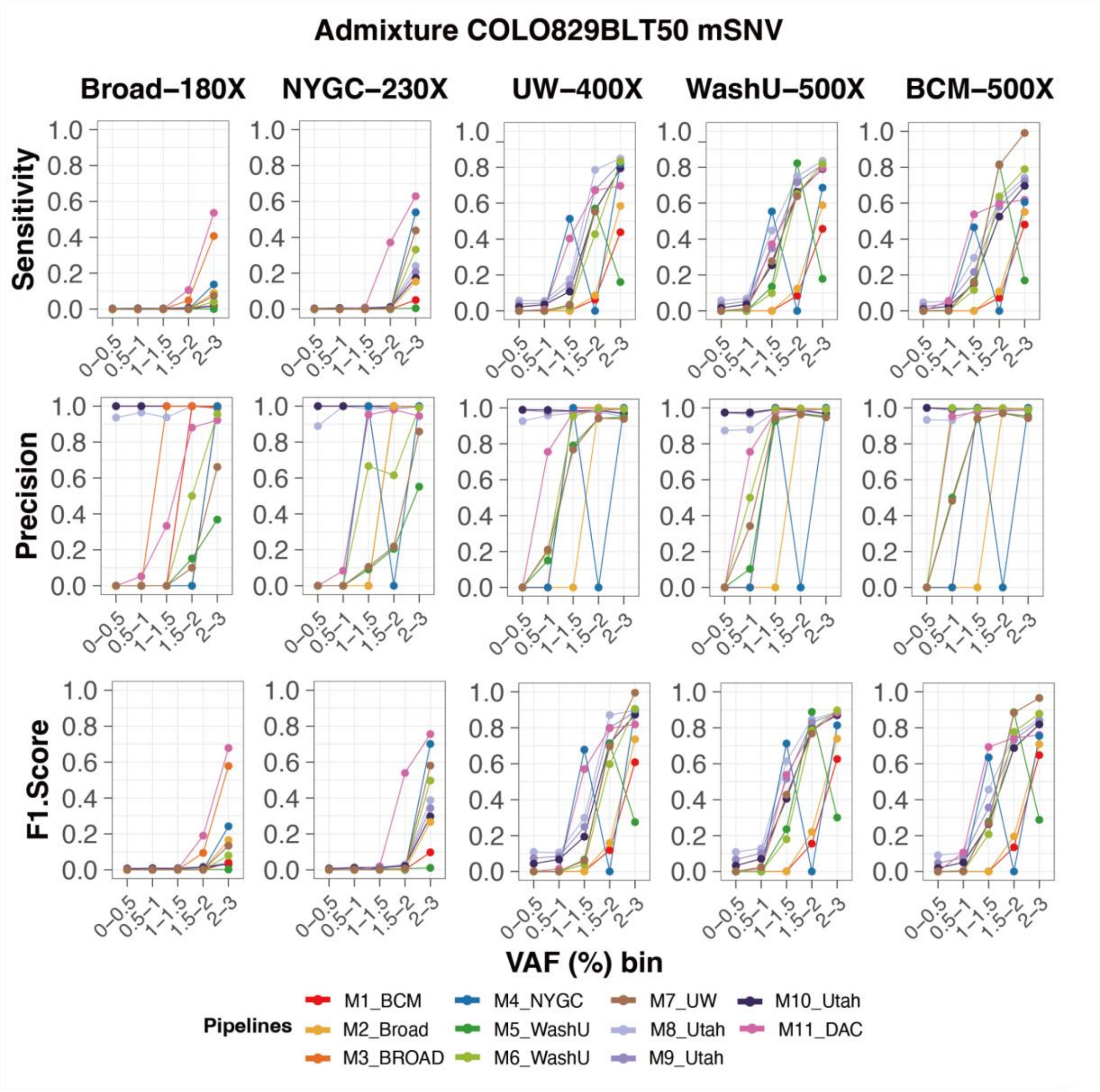

Figure S5. Performance evaluation of the mosaic SNVs with cell admixture COLO829BLT50. Sensitivity, Precision, and F1 score are shown with five independently sequenced COLO829BLT50. Ten or eleven detection pipelines were applied (**Table S3**) for mosaic SNVs in VAF below 3%.

bioRxiv preprint doi: https://doi.org/10.1101/2025.10.13.681545; this version posted October 14, 2025. The copyright holder for this preprint (which was not certified by peer review) is the author/funder, who has granted bioRxiv a license to display the preprint in perpetuity. It is made available under aCC-BY 4.0 International license.

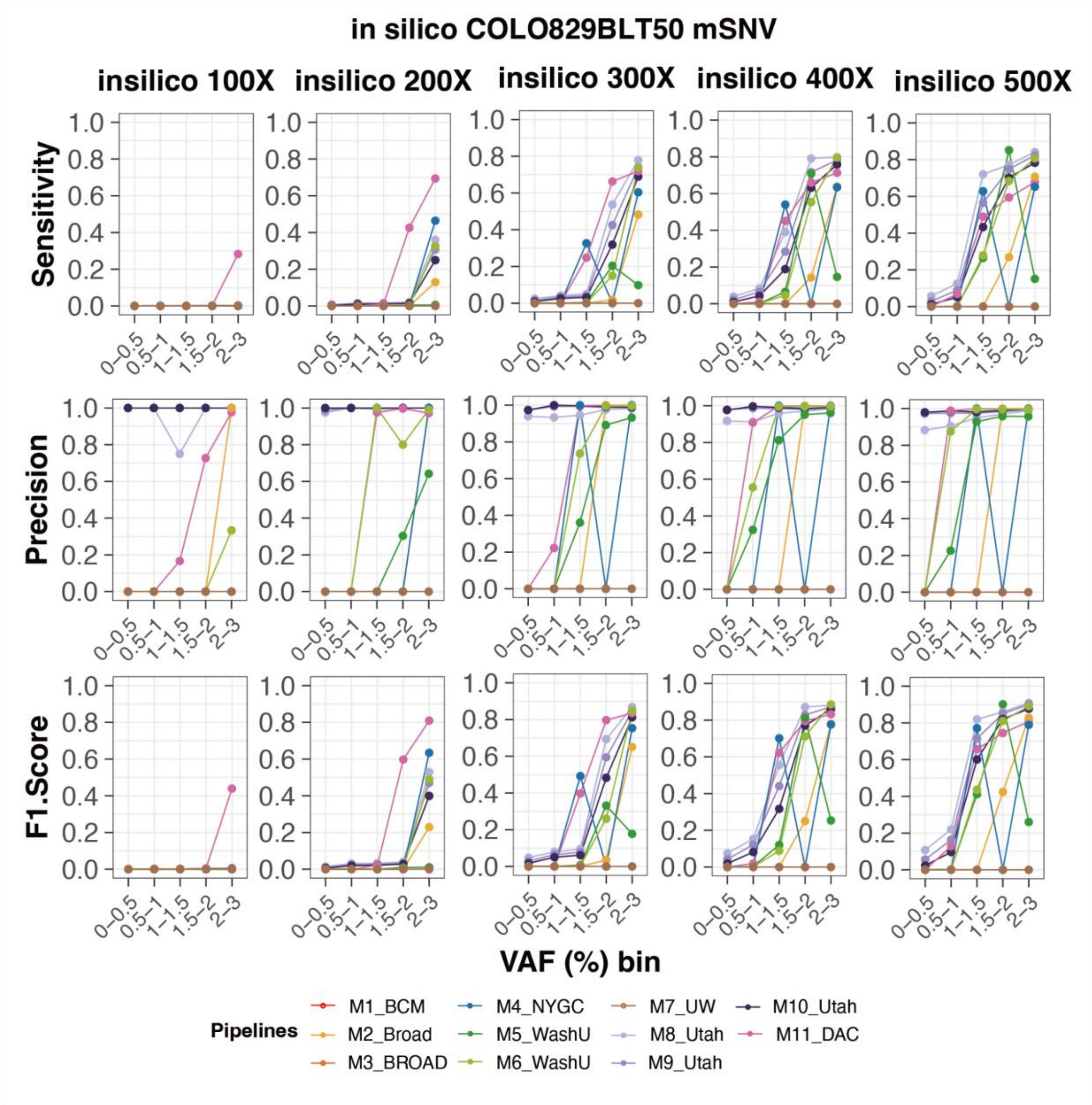

Figure S6. Performance evaluation of the mosaic SNVs with *in silico* COLO829BLT50. Sensitivity, Precision, and F1 score are shown with computationally generated COLO829BLT50 in five sequencing depths, ranging from 100× to 500×. Nine detection pipelines were applied (**Table S3**) for mosaic SNVs in VAF below 3%.

bioRxiv preprint doi: https://doi.org/10.1101/2025.10.13.681545; this version posted October 14, 2025. The copyright holder for this preprint (which was not certified by peer review) is the author/funder, who has granted bioRxiv a license to display the preprint in perpetuity. It is made available under aCC-BY 4.0 International license.

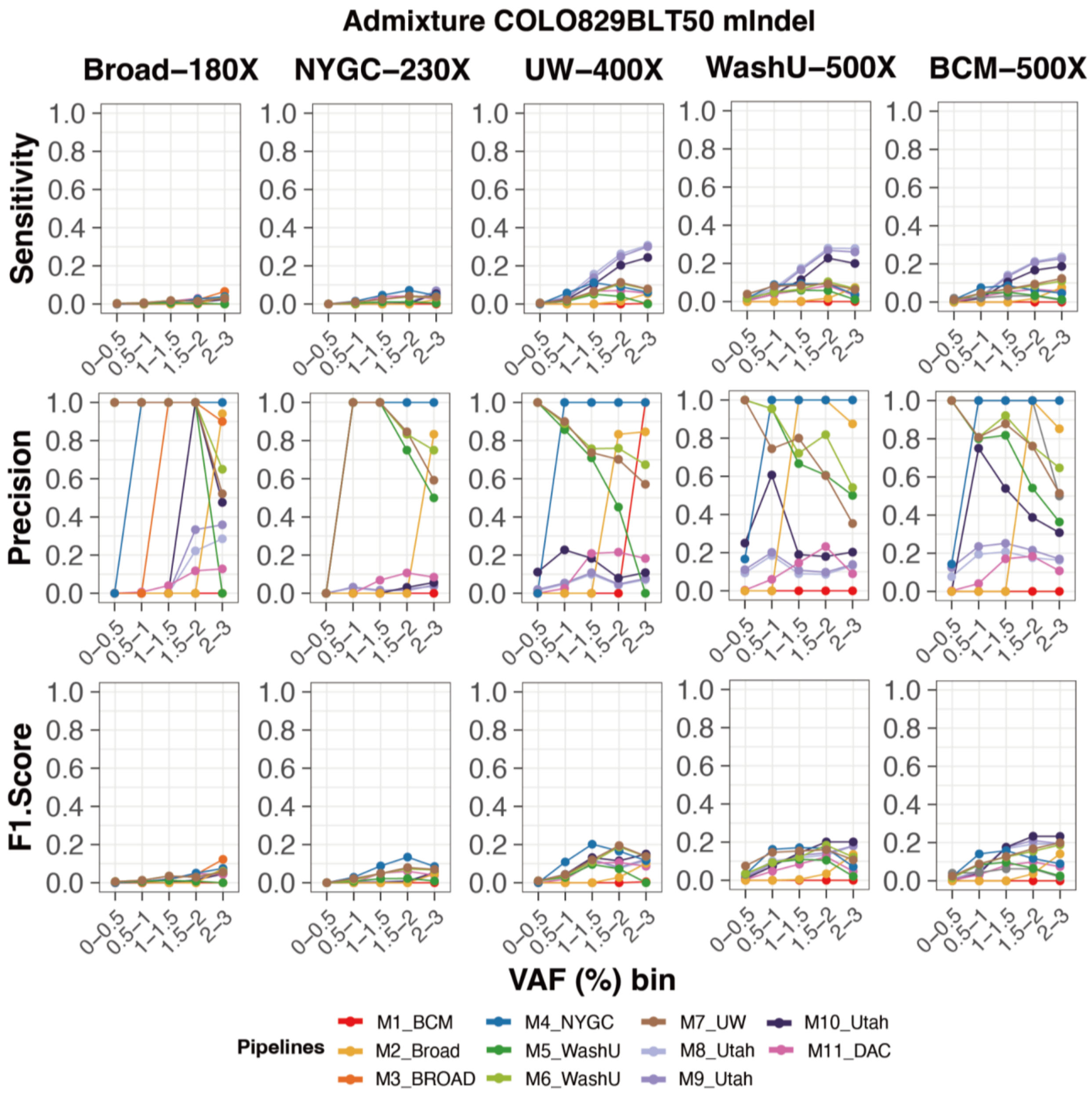

Figure S7. Performance evaluation of the mosaic Indels with cell admixture COLO829BLT50. Sensitivity, Precision, and F1 score are shown with five independently sequenced COLO829BLT50. Ten or eleven detection pipelines were applied (**Table S3**) for mosaic Indels in VAF below 3%.

bioRxiv preprint doi: https://doi.org/10.1101/2025.10.13.681545; this version posted October 14, 2025. The copyright holder for this preprint (which was not certified by peer review) is the author/funder, who has granted bioRxiv a license to display the preprint in perpetuity. It is made available under aCC-BY 4.0 International license.

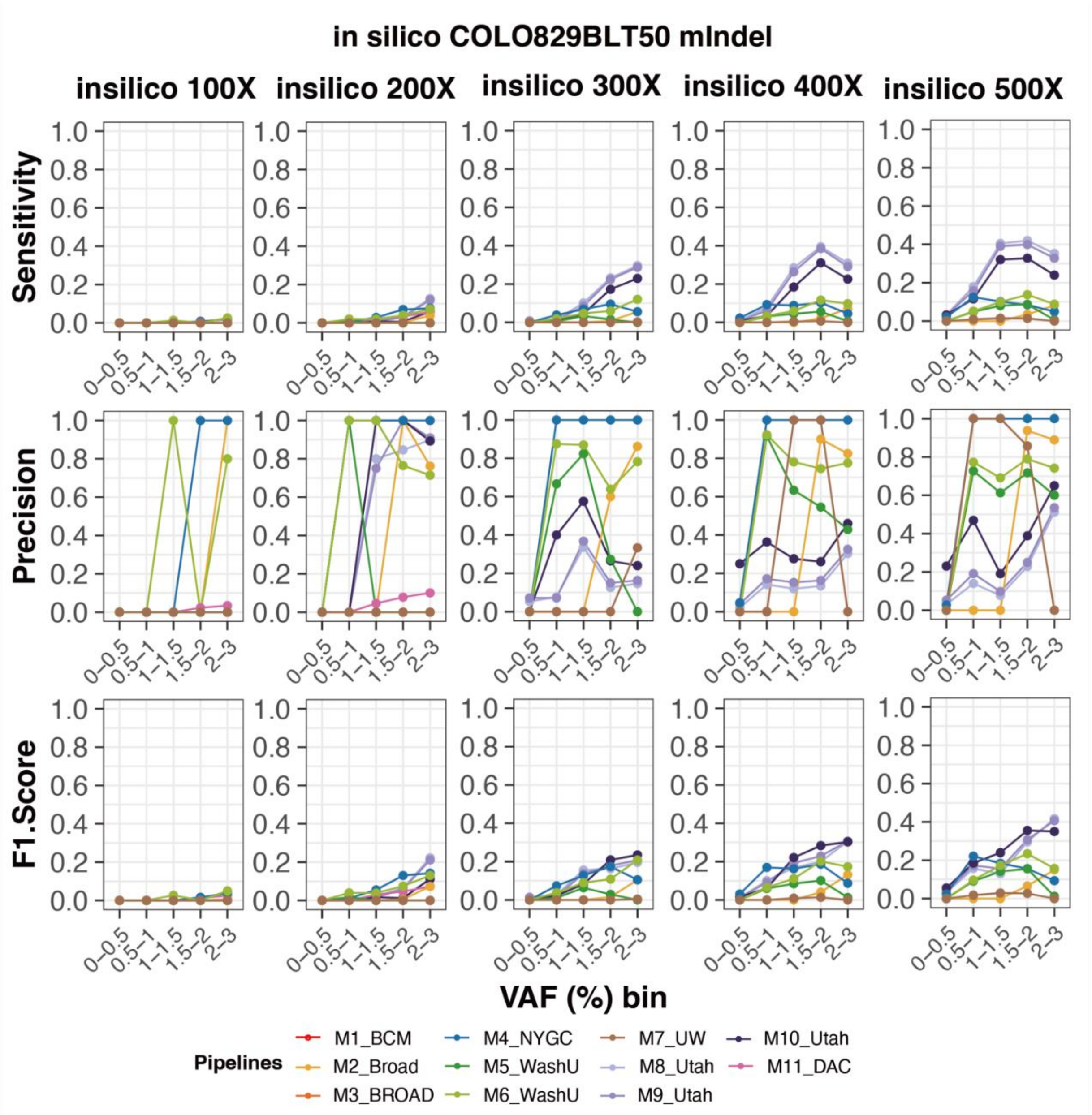

Figure S8. Performance evaluation of the mosaic Indels with *in silico* COLO829BLT50. Sensitivity, Precision, and F1 score are shown with five independently sequenced COLO829BLT50. Nine detection pipelines were applied (**Table S3**) for mosaic SNVs in VAF below 3%.

bioRxiv preprint doi: https://doi.org/10.1101/2025.10.13.681545; this version posted October 14, 2025. The copyright holder for this preprint (which was not certified by peer review) is the author/funder, who has granted bioRxiv a license to display the preprint in perpetuity. It is made available under aCC-BY 4.0 International license.

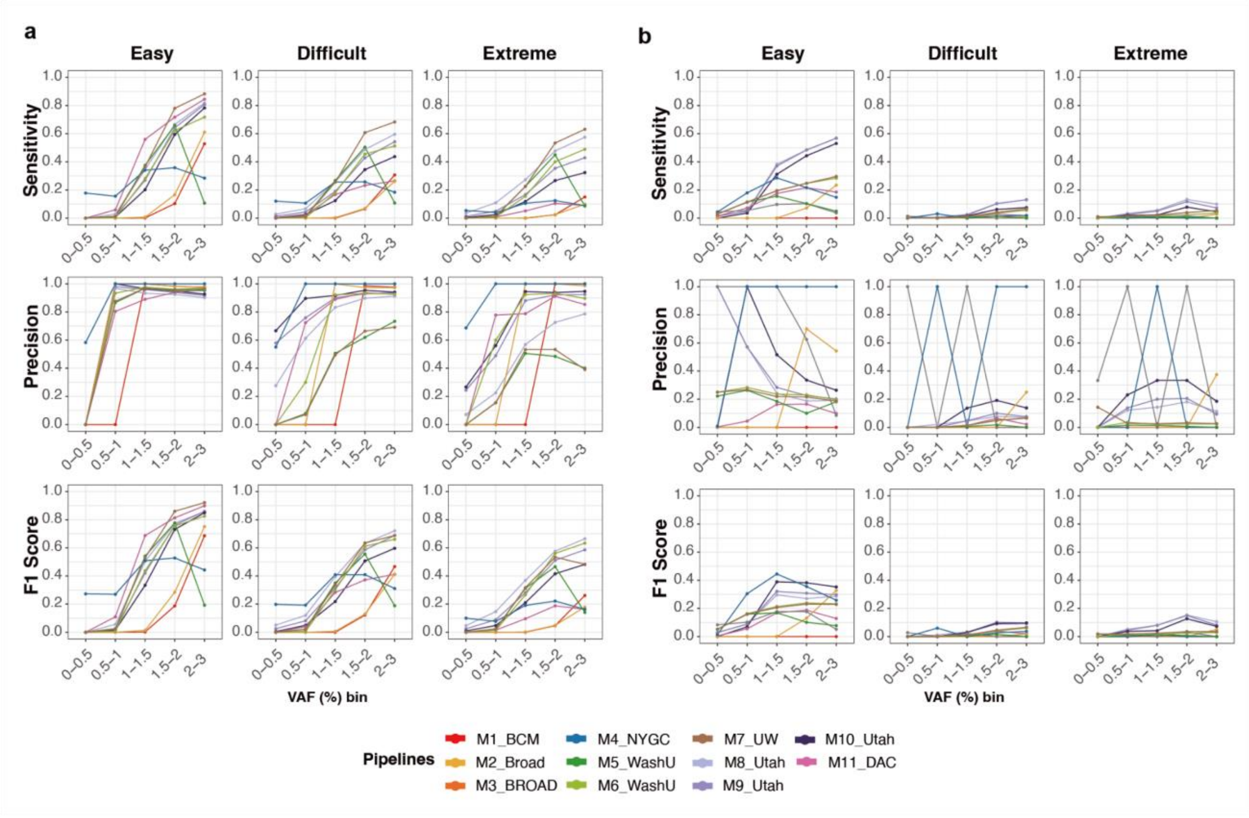

Figure S9. Performance evaluation of the mosaic SNVs and Indels in different genomic contexts. Sensitivity, Precision, and F1 scores are shown for ultra low-VAF (a) mSNVs and (b) mIndels. COLO829BLT50 with 500× coverage data was used, generated by BCM. Ten different pipelines were evaluated across easy, difficult, and extreme regions.

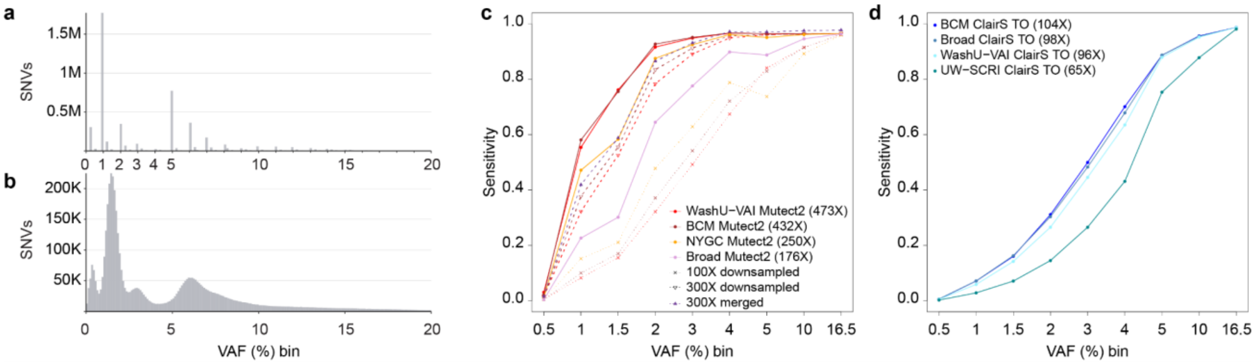

Figure S10. Expected and observed VAF distribution and detailed sensitivity in HapMap samples. Distribution of expected VAFs (a) and observed VAFs (b) in the HapMap mixture, as calculated by pileup at target positions. (c) Sensitivity of somatic variant calling with Mutect2 across expected VAF bins in Illumina short-read HapMap samples, including datasets downsampled to 300× and 100× coverage, as well as merged to 300×.(d) Sensitivity of somatic variant calling with ClairS TO across expected VAF bins per PacBio long-read sample.

bioRxiv preprint doi: https://doi.org/10.1101/2025.10.13.681545; this version posted October 14, 2025. The copyright holder for this preprint (which was not certified by peer review) is the author/funder, who has granted bioRxiv a license to display the preprint in perpetuity. It is made available under aCC-BY 4.0 International license.

Reference

1. Danecek, P., Bonfield, J.K., Liddle, J., Marshall, J., Ohan, V., Pollard, M.O., Whitwham, A., Keane, T., McCarthy, S.A., Davies, R.M., et al. (2021). Twelve years of SAMtools and BCFtools. Gigascience *10*. https://doi.org/10.1093/gigascience/giab008.

2. Broad Institute Picard Tools. http://broadinstitute.github.io/picard/.

3. Pedersen, B.S., and Quinlan, A.R. (2018). Mosdepth: quick coverage calculation for genomes and exomes. Bioinformatics *34*, 867–868. https://doi.org/10.1093/bioinformatics/btx699.

4. Andrews, S. (2010). FastQC - A quality control tool for high throughput sequence data. Babraham Bioinformatics. http://www.bioinformatics.babraham.ac.uk/projects/fastqc/.

5. The Somatic Mosaicism across Human Tissues Network Comprehensive benchmarking of somatic mutation detection by the SMaHT Network.

Table S1: Overview of sequencing depth of short-read and long-read datasets used for analysis of COLO289 and HapMap mixture benchmarking experiments.

Sample	Center	Illumina short-read WGS	PacBio long-read WGS
**COLO829**	UW-SCRI-GCC	367.9X	168.4X
	**TOTAL**	**367.9X**	**168.4X**
**COLO829BL**	UW-SCRI-GCC	277.9X	325.5X
	**TOTAL**	**277.9X**	**325.5X**
**COLO829BLT50**	BCM-GCC	474.9X	106.4X
	Broad-GCC	166.6X	156.5X
	NYGC-GCC	213.6X	--
	UW-SCRI-GCC	385.4X	23.8X
	WashU-VAI-GCC	471.5X	102.7X
	**TOTAL**	**1,712.0X**	**389.5X**
**HapMap mixture**	BCM-GCC	432.5X	103.6X
	Broad-GCC	167.2X	98.1X
	NYGC-GCC	250.0X	--
	UW-SCRI-GCC	--	65.4X
	WashU-VAI-GCC	473.1X	95.6X
	**TOTAL**	**1,322.8X**	**362.7X**

Table S2: PacBio validation rate of short-read-based SNV and Indel calls in COLO829BL for different variant calling tools.

Method	Mutation type	Total Illumina call	Alt in COLO829BL	No alt in COLO829	Valid	Valid rate (%)	No alt in COLO829 rate (%)	Alt in COLO829BL rate (%)
**Mutect2**	SNV	44,623	1,349	624	42,476	95.19	1.40	3.02
**Strelka2**		55,673	5,608	5,642	43,716	78.52	10.13	10.07
**RUFUS**		38,790	993	380	37,316	96.20	0.98	2.56
**VarNet**		44,288	1,114	3,208	39,693	89.62	7.24	2.52
**Mutect2**	Indel	2,416	35	650	1,675	69.33	26.90	1.45
**Strelka2**		2,095	134	767	1,194	56.99	36.61	6.40
**RUFUS**		2,099	355	714	1,008	48.02	34.02	16.91
**VarNet**		800	6	65	729	91.13	8.13	0.75

Table S3: Variant calling methods applied to *in-silico* and cell-admixture COLO829BLT50 samples by participating institutions.

Method		Institution	Indel	*In-silico* COLO829BLT50					Cell-admixture COLO829BLT50				
				100X	200X	300X	400X	500X	BCM	Broad	NYGC	UW-SCRI	WashU-VAI
**M1**	DRAGEN	BCM	Yes										
**M2**	DRAGEN		Yes										
**M3**	Direct DRAGEN	Broad	Yes										
**M4**	Lancet2	NYGC	Yes										
**M5**	Mutect2-VAF-0.2		Yes										
**M6**	Mutect2-dbSNP	WashU-VAI	Yes										
**M7**	Mutect2	UW-SCRI	Yes										
**M8**	RUFUS-Strict		Yes										
**M9**	RUFUS-Moderate		Yes										
**M10**	RUFUS-Lenient	Utah (*uses blood control)	Yes										
**M11**	MosaicForecast	DAC	Yes										
**Total # of variant call sets**				9	9	9	9	9	10	11	10	10	10

**SNV call set submisson status**
Provided
Not contributed
sequenced at the same

Table S4: F1 Score average across different VAF ranges and sequencing depths.

	Sequencing Depth				
	**100x**	**200x**	**300x**	**400x**	**500x**
**VAF < 1%**	0	0	0.00024531	0.00967606	0.06414392
**1% < VAF < 3%**	0.14807572	0.476122	0.67649267	0.74948148	0.73641945
**gain VAF < 1%**	NA	0	0.00024531	0.00943075	0.05446786
**gain 1% < VAF < 3%**	NA	0.32804628	0.20037068	0.07298881	-0.013062
**Gain percentage in VAF < 1%**	NA	NA	NA	38.44421344	5.629136239
**Gain percentage in 1% < VAF < 3**	NA	2.215395475	0.420838945	0.10789298	-0.017428049

Table S5: Population code and number of germline SNVs within confident regions per individual HapMap cell line contributing to the HapMap mixture.

HapMap cell line	Population code	Number of SNVs
HG002	EUR/ASH JEW	3,176,093
HG005	CHB	3,143,272
HG00438	EAS	3,168,150
HG02257	AFR	3,816,806
HG02486	AFR	3,843,750
HG02622	AFR	3,886,058
